# Impact of Peritoneal Dialysis Treatment on Arterial Stiffness and Vascular Changes in Diabetic Type 2 and Nondiabetic Patients with End-Stage Renal Disease

**DOI:** 10.1155/2013/681454

**Published:** 2013-10-22

**Authors:** Damir Rebić, Senija Rašić, Velma Rebić

**Affiliations:** ^1^Clinic for Nephrology, Clinical Center of Sarajevo University, Bolnička 25, 71000 Sarajevo, Bosnia and Herzegovina; ^2^Faculty of Medicine, University of Sarajevo, Čekaluša 90, 71000 Sarajevo, Bosnia and Herzegovina

## Abstract

Diabetes mellitus (DM) is the leading cause of the end-stage renal disease (ESRD). Vascular diseases are the most common cause of morbidity and mortality in the chronic kidney disease. The aim of this study was to analyze the impact of peritoneal dialysis (PD) treatment on morphologic and hemodynamic vascular parameters of carotid arteries in diabetic type 2 and nondiabetic patients with ESRD during the period of one year after the start of PD treatment using ultrasonography of carotid arteries and their relation on uremia and PD inherent factors. Mean intima-media thickness, plaque score, peak systolic velocity, end-diastolic velocity, and carotid diameter significantly decreased 12 months after PD treatment start in both groups. Significant reduction in median serum endothelin-1 concentration after 12 months on PD treatment was observed in the group of patients with DM (7.6–5.9 pg/mL) and also in group of patients without DM (3.6–3.3 pg/mL). Also median nitric oxide concentration significantly increased after 12 months on PD compared to baseline levels both in patients with DM (25.0–34.3 **μ**mol/L) as was observed in patients without DM (49.6–56.5 **μ**mol/L). PD treatment, with the regulation of these vasoactive molecules and other vascular risk factors, significantly contributes to vascular remodeling, especially in DM patients.

## 1. Introduction

Diabetes mellitus (DM) is the leading cause of the end-stage renal disease (ESRD) in many countries of the world. In Bosnia and Herzegovina, 17.3% of dialysis patients have primarily diabetes mellitus, while in Korea as many as 44.9% dialysis patients developed ESRD as a consequence of diabetes nephropathy [1]. Although the survival rate in DM patients with ESRD partially improved, it is still significantly lower than that in general population [2]. The results of NECOSAD study showed that survival in diabetic patients with ESRD was worse compared to nondiabetic patients and that diabetes mellitus has a very strong impact on survival even if it is not the primary cause of ESRD [3].

Risk factors for cardiovascular death in these patients include those that affect the general population as well as those related to end-stage renal disease and those that are specific to peritoneal dialysis. The development of over hydration after loss of residual renal function (RRF) is probably the most important cardiovascular risk factor specific to peritoneal dialysis. The high glucose load associated with peritoneal dialysis may lead to insulin resistance and to the development of an atherogenic lipid profile. The presence of glucose degradation products in conventional dialysis solutions, which leads to the local formation of advanced glycation end products, is also specific to peritoneal dialysis [4].

Although nonatherosclerotic cardiovascular diseases, like volume overload and left ventricular hypertrophy, can mostly contribute to the high prevalence of mortality in peritoneal dialysis (PD) patients, the recent studies suggest that PD patients are especially susceptible to the accelerated atherosclerotic process [5, 6]. Peritoneal dialysis is increased with higher frequency of several traditional (arterial hypertension, diabetes mellitus, and hyperlipidemia) and uremia-related risk factors (anemia, hypoalbuminemia, hyperhomocysteinemia, oxidative stress, microinflammation, and secondary hyperparathyroidism), which can affect the creation of vascular changes.

Important predictors of discovery of the development of accelerated atherosclerosis in PD patients are considered to be morphologic changes on common carotid arteries (CCA) in the form of the intima-media thickness (IMT) and hemodynamic changes expressed through parameters—peak systolic velocity (PSV), end-diastolic velocity (EDV), and CCA diameter to the aim of the assessment of the stage of stenosis on CCA [7, 8].

In this study, we explored the impact of PD on atherosclerotic process in patients with and without DM, monitored through morphologic and hemodynamic parameters on CCA in ESRD patients during the period of one year after the start of PD treatment. Also, the aim of the study is to establish factors that predict changes on carotid arteries as a surrogate atherosclerosis in patients with DM on PD.

## 2. Materials and Methods

### 2.1. Patients

This prospective longitudinal study includes 50 ESRD patients (diabetic type 2 and nondiabetic) who were treated with continuous ambulatory PD (CAPD) at the Clinic for Nephrology of the Clinical Center of Sarajevo University and who were observed for a year after the commencement of dialysis treatment. All examined patients underwent 4 to 5 dialysis changes with 2 liters of dialysis solution.

The patients with the verified diagnosis of carotid artery stenosis, heart valve diseases, and cerebral vascular diseases, as well as with signs of peritonitis during the study period, were excluded from the study. In all patients' antihypertensive therapy, as well as therapy (e.g., nitrites, sildenafil, captopril, NSAID's, nitroprusside, etc.) that can influence the values of monitored laboratory parameters were excluded 24 hours before taking blood samples for determination of concentration of endothelin-1 (Et-1) and nitric oxide (NO). The patients were free from taking diuretic therapy prior to diuresis level measurement and sampling.

The baseline of laboratory findings with determination of Et-1 and NO in serum complemented with ultrasonography of carotid arteries at the very beginning of dialysis treatment and after one year.

The study was conducted with the approval of the Local Ethics Committee and with the respect for the rules of ethical principles in medical research. All patients gave their informative consent for the participation in the study.

### 2.2. Laboratory Measurements

The baseline of laboratory findings was done by standard laboratory procedures at the Institute for Clinical Chemistry and Biochemistry, Clinical Center of Sarajevo University. The measurement of serum concentration of Et-1 was done by the ELISA method (Enzyme immunoassay for the quantitative determination of human endothelin (1-21) in serum, kit Biomedica Medizinprodukte GmbH & Co KG, Wien). For determination of concentration of NO in the serum, R&D System Total Nitric Oxide kit was utilized, while the concentration of NO was expressed in *μ*mol/L. Blood samples for both methods were analyzed at the Institute for Biochemistry of the Clinical Hospital *Sestre milosrdnice* in Zagreb, Croatia.

### 2.3. Measurement of Ultrasound Parameters of Carotid Artery

Intima-media thickness of common carotid artery (IMT CCA) and the presence of atherosclerotic plaque were measured by means of highly-resolute transducer sond of 7.5 MHz frequency in B mode (Wall-Track system: W-T, Maastricht, the Netherlands) from the angiologist who was not familiar with the clinical status of the study patients. The CCA, carotid bulbus, and the first 2 cm of the internal and external carotid artery were scanned, bilaterally. The CCA IMT measurements were done in the plaque-free segment. The carotid plaques were defined and counted as silent grey echo's (soft plaques) or bright white echo's (calcified plaques) that protrude into the lumen of the artery. The measurements of IMT were done at the distance of 20 mm from bifurcation into the plaque-free area on CCA. Three measurements were done on the left and right CCA, while the mean values of these measurements were utilized in the analysis. Plaque score in the carotid artery was calculated by summing the three measurements IMT values, bilaterally [9].

### 2.4. Parameters of Peritoneal Dialysis

Adequacy of dialysis (*K*_*t*_/*V*_urea_) was calculated from weekly total removed urea mass by daily volume of dialysate and urine (*K*_*t*_) and divided with urea distribution volume (*V*). The distribution volume of urea was calculated by using the Watson equation [10].

Residual renal function was estimated as the mean of renal creatinine clearance (mL/min). A simplified peritoneal equilibration test was performed using 4.25% glucose-based solution to obtain the dialysate to plasma creatinine concentration ratio at 4 hours of dwell (D/P Cr). Patients were categorized as high, high-average, and low-average transporters according to criteria of Chung and colleagues [11].

### 2.5. Statistical Analysis

All data were expressed as the mean ± SD or as median and interquartile range. The distribution of variables was tested by the Shapiro-Wilk test. Significant change in the variables from baseline to 12 months after treatment was tested by paired *t*-test for the variables that followed normal distribution or by the Wilcoxon signed-rank test for the variables that had skewed distribution. The difference between two groups was analyzed by the Mann-Whitney test. A multiple regression analysis was applied to examine the relationship between ultrasound parameters of CCA and a set of clinical and laboratory parameters. The significant independent variables were ordered according to their standardized effect, defined as regression coefficient/standard error of the regression (*β*). *P* values less than 0.05 were considered statistically significant. All statistical calculations were performed with the SPSS 16 software (version 16.0, SPSS Inc., Chicago, IL, USA).

## 3. Results

Mean blood pressure and serum lipids values significantly decreased after 12 months on PD treatment both in patients with DM type 2 and in group of patients without DM. After the 12-month PD treatment, patients with DM type 2 had significantly higher blood pressure and serum lipids level compared to group of patients without DM. Although median serum CRP levels significantly decreased after PD treatments in both groups of patients, patients with DM type 2 had significantly increased median serum CRP concentration compared to patients without DM 12 months after PD treatment (Table 1).

In group of patients with DM type 2 significant reduction in mean P, CaxP, PTH, and fibrinogen levels were observed 12 months after PD compared to the values before the treatment. Mean serum albumin was significantly lower, and product CaxP, PTH, and fibrinogen levels were significantly increased in patients with DM type 2 compared to the group of patients without DM 12 months after PD treatment (Table 1).

In group of patients with DM type 2 significant reduction in mean diuresis level, serum creatinine levels were observed 12 months after PD compared to the values before the treatment. Mean diuresis values were significantly lower, while mean creatinine and RRF levels were significantly increased in patients with DM type 2 compared to the group of patients without DM 12 months after PD treatment.

In the group of patients with DM type 2, a significant higher number of patients with *K*_*t*_/*V*_urea_ < 2.0 were observed before the treatment compared to 12 months after. Also 44% of patients with DM type 2 at the beginning of the PD treatment had low-average transport characteristics, while 12 months after PD treatment start significantly fewer patients were with high transport characteristics of peritoneum (24%) (Table 2).

Mean IMT, plaque score, PSV, EDV, and CCA diameter significantly decreased 12 months on PD treatment in group of patients without and in group of patients with DM type 2. However, after 12 months on PD, mean IMT, plaque score, PSV, EDV, and CCA diameter values were still significantly higher in patients with DM type 2 compared to patients without DM (Table 3).

**Figure 1:**
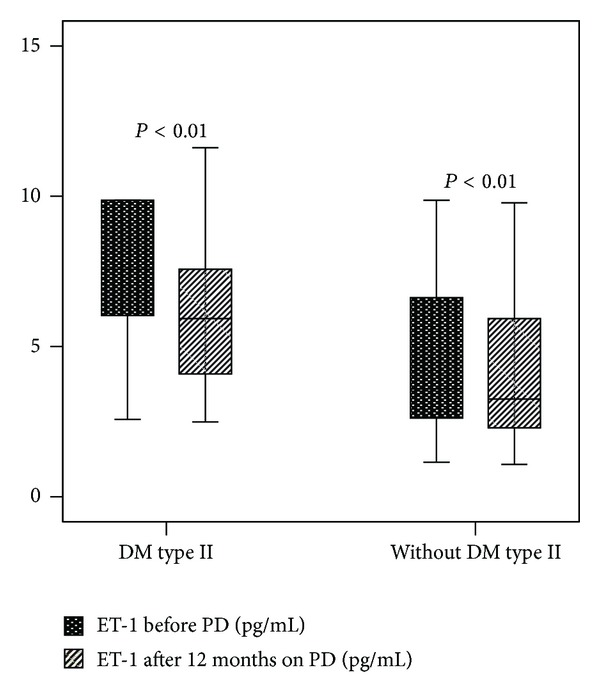
Endothelin-1 serum levels in patients with and without DM type 2 at baseline and after 12 months on peritoneal dialysis.

Median Et-1 concentration in patients with DM type 2 was significantly increased compared to median Et-1 concentration in patients without DM type 2 (7.6 (5.5–13.4) versus 3.6 (2.6–6.8) pg/mL; *P* < 0.001) before PD as well as 12 months on peritoneal dialysis (5.9 (4.1–7.8) versus 3.3 (2.3–6.0) pg/mL; *P* < 0.001). Significant reduction in median serum Et-1 concentration was observed in the group of patients with DM and without DM type 2 after 12 months of renal replacement treatment (Figure 1).

**Figure 2:**
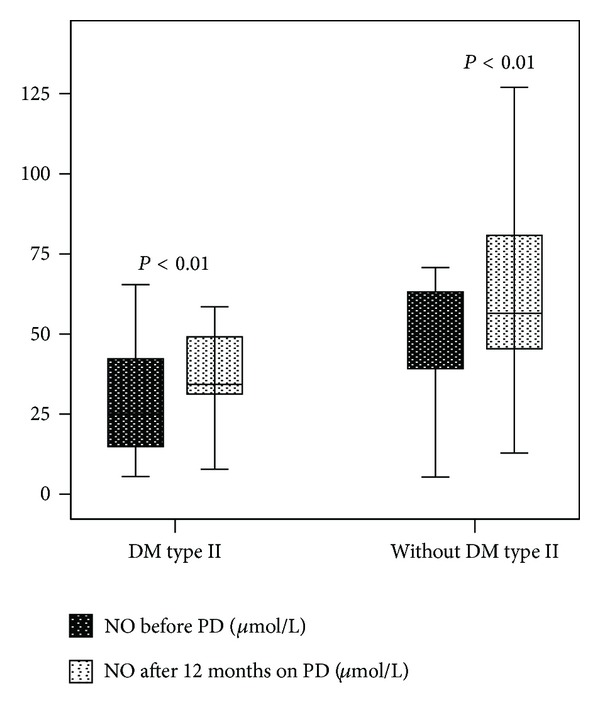
Nitric oxide serum levels in patients with and without DM type 2 at baseline and after 12 months on peritoneal dialysis.

Median NO concentration in patients with DM type 2 was significantly decreased compared to median NO concentration in patients without DM type 2 (25.0 (12.8–44.3) versus 49.6 (32.8–63.5) pg/mL; *P* < 0.001) before PD as well as 12 months on peritoneal dialysis (34.3 (26.7–49.6) versus 56.5 (44.9–89.5) pg/mL; *P* < 0.001). Significant increase in median serum NO concentration was observed in the group of patients with DM type 2 and without DM (Figure 2).

In multivariate linear regression analysis independent predictors of IMT and PSV were only serum NO level, while independent predictors of plaque score were product CaxP and Ca levels. Independent predictors of CCA diameter were serum NO, RRF, CaxP, and serum Ca levels. Independent negative predictor of EDV was NO and serum Et-1 levels were positive predictor of EDV (Table 4).

## 4. Discussion

In previous researches it was established that vascular alterations on carotid arteries can be an early sign of atherosclerosis and a predictor of generalized vascular changes in PD patients [12].

In this study we confirmed that ESRD patients at the very start of PD treatment had signs of advanced atherosclerosis, which suggests that the uremia itself is an independent risk factor for functional atherosclerotic damage. Also, in diabetic patients on PD, compared to patients without DM on PD, IMT of CCA had significantly higher values. Despite these differences, other markers of atherosclerotic changes, primarily endothelial dysfunction, were of similar values and of significantly higher values in diabetic patients. 

Blood vessel endothelium modulates vascular structure and tonus affecting the production of nitric oxide, which is considered to have a protective role in the creation of vascular atherosclerotic damage [13].

Endothelial dysfunction is a very important process which participates in the creation of the process of atherosclerosis and arteriosclerosis and is under the direct influence of traditional and nontraditional risk factors. To date numerous studies showed that the function of endothelium is weakened in ESRD patients and it is a predictor of cardiovascular mortality in these patients, but also in general population [13]. Hyperglycemia, insulin resistance, oxidative stress, and lipid metabolism disorder are involved in the development of endothelial dysfunction in patients with DM type 2. Recurrent hyperglycemia, with accumulation of advanced glycation end-products (AGEs), leads to chronic endothelial dysfunction, which is especially expressed in PD patients [14]. In patients on peritoneal dialysis atherosclerotic disease is associated with the presence of calcification in the wall of blood vessels and it is a response to hemodynamic humoral abnormalities in chronic uremia. The mutual mixture of traditional and nontraditional risk factors is one of the reasons for accelerated atherosclerosis in patients on PD [7]. These changes lead to the increased stiffness and the loss of elasticity of big arteries, as well as aorta and CCA [15].

In our study the verified increased Et-1 values in serum of uremic patients before the start of dialysis treatment may be a consequence of the reduced elimination of Et-1 from circulation due to ESRD and the damage of vascular endothelial cells. The concentration of serum endothelin-1 was significantly higher in PD patients with DM type 2, compared to nondiabetic patients, which can indicate more expressive damage of endothelium in this group of patients. It was established that hyperproduction of Et-1, accompanied by increased concentration of circulating Et-1 in dialysis patients, may lead to the development of endothelial dysfunction, with a high risk of cardiovascular mortality and morbidity in this population of patients [16].

The increased mean value of IMT and the plaque score on CCA in our patients, with hemodynamic changes on the flow of blood on CCA at the very start of peritoneal dialysis, speaks about the significant presence of morphologic and functional macrovascular changes in these patients. The mentioned results agree with the previous reports by other authors and thereby support the presumption about the “accelerated atherogenesis” even in PD patients [17] and suggest a positive effect of peritoneal dialysis on the process of vascular remodeling. 

Using multivariate analysis we found a statistically significant influence of CaxP product and the serum calcium on plaque score CCA. Hypercalcaemia and the increased product of CaxP in PD patients are linked to the development of CCA calcification, especially in diabetic patients, independent of parathormone values [18].

This study did not show the dependence of changes on carotid arteries from transport characteristics of peritoneum, which is in accordance with the research of Rodrigues and associates [19], who ascertained that the increased mortality of PD patients does not depend on peritoneal permeability but is probably the result of associated comorbidity, basic atherosclerosis, volume overload, chronic inflammation, and humoral abnormalities in uremia. The same author concluded that peritoneal transport characteristics are primarily dependent on the vasoactive intermediate compounds concentration, produced by mesothelium [20].

The role of the RRF is especially important in PD patients. The decrease in RRF is closely related to the volume overload, anemia, inflammation, malnutrition, and eventually increased morbidity. 

The reduction of creatinine clearance is very much associated with endothelial dysfunction and arterial rigidity [21] in patients with chronic kidney disease, which indicates that the function of kidneys plays an important role in the protection of blood vessels. In our results, we concluded that RRF appeared as an independent predictor of CCA diameter but not other morphological and functional parameters of CCA in diabetic patients.

## 5. Conclusion

ESRD patients on PD have significant morphologic and hemodynamic changes on CCA before the start of dialysis treatment. Patients with DM type 2 have significantly higher values of IMT CCA in relation to nondiabetic patients on PD. The results of this study suggest the association of NO concentration in serum with significant morphological and functional changes on CCA, which are considered surrogate-generalized atherosclerosis in DM type 2 patients but also in non-DM PD patients, which in turn gives this biomarker the importance of a predictor of the development of accelerated atherosclerosis in this patients. 

The treatment with peritoneal dialysis, with the regulation of these vasoactive molecules and other vascular risk factors, importantly impedes vascular remodeling, especially in DM type 2 patients. Intensive monitoring of hemodynamic and nonhemodynamic vascular risk factors and vascular parameters in PD patients is necessary for the purpose of prevention and even regression of undesired changes on vascular system.

## Figures and Tables

**Table 1:** Baseline characteristics of the patients before and after 12 months on PD treatment.

Variable	Without DM		With DM type 2	
	Basal	After 12 months	Basal	After 12 months
Age (y)	50.9 ± 17.6		58.3 ± 13.9	
Smoking	11 (44.0%)		13.0 (52.0%)	
Males	11 (44.0%)		14.0 (56.0%)	
SBP (mmHg)	143.0 ± 19.0	130.8 ± 13.2*	152.0 ± 20.4**	137.6 ± 15.1^∗†^
DBP (mmHg)	85.6 ± 11.9	75.6 ± 8.2*	92.8 ± 12.4**	83.2 ± 9.9^∗†^
BMI (kg/m^2^)	24.8 ± 3.4	24.9 ± 2.5	27.1 ± 3.6**	26.6 ± 2.5^†^
Cholesterol (mmol/L)	5.7 ± 1.1	5.3 ± 0.8*	7.3 ± 1.7**	6.53 ± 1.2^∗†^
Triglyceride (mmol/L)	2.0 ± 1.3	1.58 ± 0.4*	2.8 ± 1.2**	1.9 ± 0.3^∗†^
Albumin (g/L)	32.9 ± 4.5	31.3 ± 2.3	28.5 ± 3.3**	30.5 ± 2.8*
Ca	2.2 ± 0.13	2.2 ± 0.1	2.2 ± 0.21	2.24 ± 0.1
P	1.7 ± 0.3	1.57 ± 0.24*	1.82 ± 0.3**	1.7 ± 0.2*
CaxP	3.8 ± 0.68	3.5 ± 0.62*	4.0 ± 0.54	3.8 ± 0.6^∗†^
PTH	125 (87–267.5)	130 (88.6–210)	259 (181.6–430.0)**	310 (177.5–429.5)^∗†^
Fibrinogen	5.6 ± 2.1	4.4 ± 1.5*	6.7 ± 1.5**	5.0 ± 1.1^∗†^
CRP (mg/dL)	7.3 (3.9–11.9)	3.9 (3.2–6.3)*	13.2 (9.5–21)**	8.1 (5.3–10.5)^∗†^

*Significant difference in the same group before (basal) and 12 months after PD treatment.

**Significant difference between groups before the PD treatment.

^†^Significant difference between groups 12 months after PD treatment.

SBP: systolic blood pressure; DBP: diastolic blood pressure; BMI: body mass index; Ca: calcium; P: phosphorous; PTH: parathyroid hormone; CRP: C reactive protein.

**Table 2:** Parameters of peritoneal dialysis before and after 12 months of treatment in patients with DM type 2 and without of DM.

Variable	Without DM		With DM type 2	
	Basal	After 12 months	Basal	After 12 months
Diuresis (mL)	626.0 ± 383.0	714.0 ± 479.3*	465 ± 363.9**	362.0 ± 273.6^∗†^
RRF (mL/min)	6.1 ± 3.8	8.0 ± 3.9*	4.9 ± 3.2**	4.9 ± 3.9^†^
Creatinine (*μ*mol/L)	838.5 ± 253.2	714.5 ± 154.0*	986.2 ± 162.3**	815.2 ± 109.3^∗†^
*K*_*t*_/*V*_urea_ < 1.7	3 (12.0%)		1 (4.0%)	2 (8.0%)
*K*_*t*_/*V*_urea_ 1.7–2.0	3 (12.0%)	4 (16.0%)	18 (72.0%)	14 (56.0%)
*K*_*t*_/*V*_urea_ > 2.0	19 (76.0%)	21 (84.0%)	6 (24.0%)**	9 (36.0%)^†^
Low-average transporters (T)	23 (92.0%)	24 (96.0%)	11 (44.0%)	11 (44.0%)
High-average T	1 (4.0%)	1 (4.0%)	10 (40.0%)	8 (32.0%)
High T			4 (16.0%)**	6 (24.0%)^†^

*Significant difference in the same group before (basal) and 12 months after PD treatment.

**Significant difference between groups before the PD treatment.

^†^Significant difference between groups 12 months after PD treatment.

RRF: residual renal function.

**Table 3:** Ultrasound parameters of CCA.

Parameters	Without DM		With DM type 2	
	Basal	After 12 months	Basal	After 12 months
IMT (mm)	0.62 ± 0.15	0.54 ± 0.16*	0.9 ± 0.2**	0.85 ± 0.18^∗†^
Plaque score	3.57 ± 0.97	3.2 ± 0.92*	5.2 ± 1.0**	4.7 ± 1.1^∗†^
PSV (cm/s)	118.0 ± 18.7	100.3 ± 20.4	153.7 ± 27.8**	130.4 ± 24.0^∗†^
EDV (cm/s)	37.9 ± 14.7	35.2 ± 11.2*	60.6 ± 19.6**	53.4 ± 18.7^∗†^
CCA diameter (mm)	5.67 ± 0.65	5.32 ± 0.65*	6.1 ± 0.9**	5.8 ± 0.7^∗†^

*Significant difference in the same group before (basal) and 12 months after PD treatment.

**Significant difference between groups before the PD treatment.

^†^Significant difference between groups 12 months after PD treatment.

IMT: intima-media thickness; EDV: end-diastolic velocity; PSV: peak systolic velocity; CCA: common carotid artery.

**Table 4:** Independent predictors of ultrasound parameters in patients with DM type 2 after 12 months on peritoneal dialysis.

Dependent variable	Predictors	Unstandardized coefficients		Standardized coefficients	*t*	*P*
		*B*	Standard error	Beta		
IMT (mm) (*R* ^2^ = 0.56)	NO	−0.5	0.1	−0.75	−4.8	<0.001

Plaque score	CaxP	0.9	0.27	0.5	3.2	0.005
(*R* ^2^ = 0.77)	Ca	−2.3	11	−0.3	−2.1	0.048

EDV (cm/s)	NO	−41.2	10.7	−0.6	−3.8	0.001
(*R* ^2^ = 0.81)	Et-1	21.1	9.2	0.36	2.3	0.03

PSV (cm/s) (*R* ^2^ = 0.72)	NO	−83.2	12.4	−0.85	−6.7	0.001

CCA diameter (mm) (*R* ^2^ = 0.78)	NO	−2.055	0.40	−0.8	−5.1	0.001
	RRF	0.114	0.03	0.53	4.1	<0.01
	CaxP	0.76	0.14	0.60	5.3	<0.01
	Ca	−1.7	0.7	−0.32	−2.5	<0.026

IMT: intima-media thickness; EDV: end-diastolic velocity; PSV: peak systolic velocity; CCA: common carotid artery.

NO: nitric oxide; Ca: calcium; P: phosphorous; Et-1: endothelin-1; RRF: residual renal function.
